# Differential Safety Between Top-Ranked Cancer Hospitals and Their Affiliates for Complex Cancer Surgery

**DOI:** 10.1001/jamanetworkopen.2019.1912

**Published:** 2019-04-12

**Authors:** Jessica R. Hoag, Benjamin J. Resio, Andres F. Monsalve, Alexander S. Chiu, Lawrence B. Brown, Jeph Herrin, Justin D. Blasberg, Anthony W. Kim, Daniel J. Boffa

**Affiliations:** 1 Section of Thoracic Surgery, Department of Surgery, Yale School of Medicine, New Haven, Connecticut; 2 Department of Internal Medicine, Cancer Outcomes Public Policy and Effectiveness Research Center, Yale School of Medicine, New Haven, Connecticut; 3 Department of Health Policy and Management, Yale School of Public Health, New Haven, Connecticut; 4 Section of Cardiovascular Medicine, Department of Internal Medicine, Yale School of Medicine, New Haven, Connecticut; 5 Department of Surgery, University of Southern California Keck School of Medicine, Los Angeles

## Abstract

**Importance:**

Leading cancer hospitals have increasingly shared their brands with other hospitals through growing networks of affiliations. However, the brand of top-ranked cancer hospitals may evoke distinct reputations for safety and quality that do not extend to all hospitals within these networks.

**Objective:**

To assess perioperative mortality of Medicare beneficiaries after complex cancer surgery across hospitals participating in networks with top-ranked cancer hospitals.

**Design, Setting, and Participants:**

A cross-sectional study was performed of the Centers for Medicare & Medicaid Services 100% Medicare Provider and Analysis Review file from January 1, 2013, to December 31, 2016, for top-ranked cancer hospitals (as assessed by *U.S. News and World Report*) and affiliated hospitals that share their brand. Participants were 29 228 Medicare beneficiaries older than 65 years who underwent complex cancer surgery (lobectomy, esophagectomy, gastrectomy, colectomy, and pancreaticoduodenectomy [Whipple procedure]) between January 1, 2013, and October 1, 2016.

**Exposures:**

Undergoing complex cancer surgery at a top-ranked cancer hospital vs an affiliated hospital.

**Main Outcomes and Measures:**

Risk-adjusted 90-day mortality estimated using hierarchical logistic regression and comparison of the relative safety of hospitals within each cancer network estimated using standardized mortality ratios.

**Results:**

A total of 17 300 patients (59.2%; 8612 women and 8688 men; mean [SD] age, 74.7 [6.2] years) underwent complex cancer surgery at 59 top-ranked hospitals and 11 928 patients (40.8%; 6287 women and 5641 men; mean [SD] age, 76.2 [6.9] years) underwent complex cancer surgery at 343 affiliated hospitals. Overall, surgery performed at affiliated hospitals was associated with higher 90-day mortality (odds ratio, 1.40; 95% CI, 1.23-1.59; *P* < .001), with odds ratios that ranged from 1.32 (95% CI, 1.12-1.56; *P* = .001) for colectomy to 2.04 (95% CI, 1.41-2.95; *P* < .001) for gastrectomy. When the relative safety of each top-ranked cancer hospital was compared with its collective affiliates, the top-ranked hospital was safer than the affiliates in 41 of 49 studied networks (83.7%; 95% CI, 73.1%-93.3%).

**Conclusions and Relevance:**

The likelihood of surviving complex cancer surgery appears to be greater at top-ranked cancer hospitals compared with the affiliated hospitals that share their brand. Further investigation of performance across trusted cancer networks could enhance informed decision making for complex cancer care.

## Key Points

**Question:**

Is there a difference in risk-adjusted mortality between top-ranked cancer hospitals and affiliates that share their brand?

**Findings:**

In this cross-sectional study, of the 29 228 Medicare beneficiaries undergoing complex cancer surgery between 2013 and 2016 at top-ranked hospitals and their affiliates, patients treated at affiliated hospitals had a significantly higher likelihood of 90-day mortality compared with patients at top-ranked cancer hospitals. Within 84% of cancer networks, the safety performance of the top-ranked hospital was better compared with their collective affiliates.

**Meaning:**

Complex cancer surgery at top-ranked cancer hospitals is associated with a lower risk of surgical mortality than surgery performed at their affiliated hospitals.

## Teaser

This cross-sectional study compares perioperative mortality of Medicare beneficiaries after complex cancer surgery at top-ranked cancer hospitals vs their affiliated hospitals.

## Introduction

For many patients with cancer, complex surgery represents both their best chance of cure and their greatest potential for treatment-associated harm, as major complications remain common.^1,2,3,4,5^ Previous studies have identified wide variation in the safety of complex surgical procedures for cancer across hospitals, with lethal complications occurring up to 4 times more often at low-volume or underperforming hospitals.^4,6,7,8^ Unfortunately, nearly half of complex surgical procedures for cancer take place in these higher-risk hospital environments.^7,9^ As a result, multiple attempts have been made by payers and clinicians to direct patients toward the safest hospitals for complex cancer surgery, with variable outcomes.^10,11,12^ Ultimately, individual choice for hospital care may harbor the greatest potential to align patients with the safest environments for complex cancer surgery, but would require patients to be adequately informed of their safest options.

Hospital reputation is an important factor that patients consider when choosing hospitals for complex care.^13,14^ Each hospital’s name evokes a reputation for safety and quality that becomes the hospital’s “brand.” Particularly favorable reputations, including those supported by prominent national rankings (eg, *U.S. News and World Report*), can generate positive brand recognition and influence patient choice.^14,15,16^ During the past several years, leading cancer hospitals have increasingly shared their brands with smaller hospitals through affiliations. However, this brand sharing may confound patient choice, as patients may no longer be able to distinguish individual hospital reputations for safety within cancer networks.^17,18,19^

To this point, a recent nationally representative survey found that nearly half of respondents perceived the safety of complex surgery at smaller affiliated hospitals to be identical to the safety at larger hospitals specializing in cancer care (whose brand they share).^19,20^ Furthermore, 31% of respondents thought that once a local hospital formed an affiliation with a top-ranked cancer hospital, it was no longer necessary to travel to the top-ranked hospitals to undergo complex surgery.^20^

Despite public perception, there is currently no evidence to support (or refute) assumptions of care equivalency within cancer networks. Therefore, in an effort to enhance informed decision making, we evaluated the surgical mortality of Medicare beneficiaries across hospitals participating in networks with top-ranked cancer hospitals.

## Methods

### Primary Data Source

The Centers for Medicare & Medicaid Services 100% Medicare Provider and Analysis Review File and Master Beneficiary Summary File were analyzed from January 1, 2012 to December 31, 2016 (data from 2012 were used exclusively to establish preoperative comorbidities). The study was approved by the Yale Human Investigations Committee, with patient consent waived because data were deidentified. This study followed the Strengthening the Reporting of Observational Studies in Epidemiology (STROBE) reporting guideline.

The study included patients older than 65 years with a diagnosis of primary cancer of the colon, lung, pancreas, stomach, or esophagus and who underwent nonemergency complex cancer surgery (pulmonary lobectomy, colectomy, gastrectomy, pancreaticoduodenectomy [Whipple procedure], or esophagectomy) between January 1, 2013, and October 1, 2016 (eTable 1 in the Supplement).

### Hospital Selection

#### Top-Ranked Hospitals Specializing in Cancer Care

A key objective of this study was to evaluate a cohort of prominent hospitals recognized by the general public for excellence in cancer care, whose hospital brands have the greatest potential to influence patient choice for care. The study focused on hospitals ranked among the top 50 best hospitals for cancer by *U.S. News and World Report* at least once between 2013 and 2016 (n = 59). The *U.S. News and World Report* hospital rankings were chosen because reputation is a major component of their ranking method,^21^ these rankings are the most frequently advertised by larger hospitals,^14,15^ and these rankings are known to influence patient choice for care.^14,15^ Several other publicly available reports of “best cancer hospitals” are derived from *U.S. News and World Report* rankings (eg, Medscape, CNN, Livestrong, and *Men’s Health*), further highlighting the influence of our top-ranked cohort.

#### Affiliates of Hospitals Specializing in Cancer Care

Two steps were taken to establish affiliation with a top-ranked cancer hospital in a way that might influence patient choice (eFigure 1 in the Supplement). First, the American Hospital Association Annual Survey Database was queried from 2012 to 2015 to identify hospitals that participated in a network with a top-ranked cancer hospital. This step identified 637 candidate affiliates. Second, it was established that the name of the top-ranked cancer hospital was publicly associated with the affiliated hospital (brand sharing), as opposed to more restricted relationships that were not strategically promoted (ie, financial only). Each candidate affiliate was evaluated for online evidence (advertising and website) of brand sharing. A total of 388 affiliated hospitals were identified as brand sharing with a top-ranked cancer hospital, of which 343 performed complex cancer surgery during the study period (eAppendix in the Supplement).

### Outcomes

Ninety-day mortality was selected as the primary outcome as it is considered to be the most accurate measure of surgery-associated mortality.^6,22,23,24^ The Master Beneficiary Summary File was used to derive all-cause mortality occurring within 90 days of the index surgery. However, because 30-day mortality may encompass distinct elements of hospital care (eg, failure to rescue from complications), analyses were repeated using 30-day mortality. Results of these sensitivity analyses were consistent with the primary results (eTable 2 in the Supplement).

### Statistical Analysis

Two complementary approaches were used to determine the extent to which the safety of complex surgical care varied according to status within networks. The primary approach compared all patients who underwent surgery at an affiliated hospital with all patients who underwent surgery at a top-ranked hospital, which allowed for assessment of an overall affiliation association. The second approach was designed to compare associations within each hospital network and allowed for the assessment of whether safety at an affiliated hospital vs a top-ranked hospital varied across networks.

For the first analysis, hierarchical multivariable logistic regression models were estimated overall and for each procedure to evaluate the association between undergoing surgery at an affiliated hospital vs at a top-ranked cancer hospital and 90-day surgical mortality. Models included a dichotomous indicator for whether patients underwent surgery at an affiliated hospital or a top-ranked cancer hospital and included a hospital-specific random effect to account for clustering of patients within hospitals. Models were adjusted for patient characteristics including age, sex, race/ethnicity, year of surgery, Elixhauser comorbidities,^25,26^ procedure, and type of admission. The overall model further accounted for the type of procedure; procedure-specific models for colectomy and gastrectomy included further adjustment for partial vs total resection. The study period included a transition from the *International Classification of Diseases, Ninth Revision* to the *International Statistical Classification of Diseases and Related Health Problems, Tenth Revision*, which was incorporated into all diagnosis and procedures coding algorithms, including for Elixhauser comorbidities.^27^

For the second analysis, each top-ranked cancer hospital was compared with its collective set of affiliates using standardized mortality ratios (SMRs). Standardized mortality ratios were calculated as the ratio of observed to expected 90-day mortality rates. Expected mortality rates were generated from procedure-specific multivariable logistic regression models, adjusted for patient variables listed above, using all eligible beneficiaries (ie, not restricted to the top-ranked cancer hospitals and their affiliates; n = 109 635) to avoid endogeneity. A minimum volume of 10 surgical procedures was imposed to calculate SMR (by collective affiliates or by the top-ranked hospital) to reduce variation introduced by particularly low-volume hospitals.^28^ Paired *t* tests weighted by procedure volume were applied to log-transformed SMRs to distinguish top-ranked hospitals and collective affiliates from the national average. Because expected mortality was derived using all eligible beneficiaries, an SMR less than 1 indicated safer hospital performance than the national average. Within each network, the SMR of top-ranked hospitals was compared with their collective affiliates using *t* tests adjusted for multiple comparisons using the stepdown Bonferroni correction, as well as evaluation of overlapping 95% CIs. The 95% CIs around SMRs were based on 1000 bootstrapped samples.

#### Contribution of Hospital Attributes to Differential Safety

Multiple hospital-level characteristics were individually added to logistic regression models to assess the relative contribution of each hospital attribute on the differential 90-day mortality risk between top-ranked hospitals and affiliates.

#### Sensitivity Analyses

Several alternate analyses were performed to support the primary models:

1. Colectomy was more common than other procedures, particularly among affiliated hospitals. The 2 main analyses were repeated excluding patients who underwent colectomy (eTable 3 and eFigure 2 in the Supplement).
2. Two networks were particularly large, combining 23.6% of all eligible affiliated hospitals (n = 81) and 16% of patients. Adjusted hierarchical logistic regression models were estimated excluding these 2 large networks (eTable 4 in the Supplement).
3. As an alternate approach to risk adjustment, we performed reliability adjustment by estimating risk-standardized mortality ratios, which represent another metric of hospital performance used by the Centers for Medicare & Medicaid Services for quality reporting (eFigure 3 in the Supplement).^29,30^ The risk-standardized mortality ratio acts as a shrinkage estimator that will generally overestimate the performance of low-volume hospitals.^31^ We further applied reliability adjustment to compare each top-ranked cancer hospital with each of its network affiliates (eFigure 4 in the Supplement).
4. To evaluate the association between hospital rank and safety, we estimated SMRs by quintiles of the top 50 ranked hospitals (eFigure 5 in the Supplement).

The results of all sensitivity analyses were consistent with the primary results.

Comparison of categorical variables between groups was performed using χ^2^ tests and continuous parametric variables with *t* tests. All *P* values were from 2-sided tests and results were deemed statistically significant at *P* < .05. All analyses were performed using SAS, version 9.4 (SAS Institute Inc).

## Results

A total of 59 hospitals achieved a top cancer hospital ranking during the study period and were affiliated with 343 additional hospitals (Table 1).^32^ The median number of affiliates for each top-ranked hospital was 4 (interquartile range, 1-8), and 6 top-ranked hospitals had no affiliates. In general, affiliated hospitals were smaller (median number of beds, 210 [interquartile range, 148-347] vs 711 [interquartile range, 540-893]) and less likely to be teaching hospitals (38 [11.1%] vs 56 [94.9%]).

**Table 1.** Hospital Characteristics

Characteristic	Hospitals, No. (%)		*P* Value
	Affiliate (n = 343)	Top-Ranked (n = 59)	
Beds, median (IQR), No.	210 (148-347)	711 (540-893)	<.001
Commission on Cancer accredited			
Yes	230 (67.1)	58 (98.3)	<.001
No	113 (32.9)	1 (1.7)	
Teaching hospital			
Yes	38 (11.1)	56 (94.9)	<.001
No	305 (88.9)	3 (5.1)	
Duration of affiliation within study period, median (IQR), y			
≤1.0	7 (2.0)	NA	NA
1.1-2.0	64 (18.7)	NA	
2.1-3.0	46 (13.4)	NA	
>3.0	226 (65.9)	NA	
Annual volume of all procedures, median (IQR), No.^a^	10 (5-21)	74 (56-112)	<.001
Procedure volume, median (IQR), No.^b^			
Lobectomy	8 (3-18)	77 (53-107)	<.001
Colectomy	15 (7-30)	78 (59-120)	<.001
Gastrectomy	2 (1-4)	21 (15-30)	<.001
Esophagectomy	3 (1-5)	25 (12-34)	<.001
Pancreaticoduodenectomy	3 (2-8)	43 (26-62)	<.001
Proportion of colectomies performed with minimally invasive technique, median (IQR)	0.26 (0.13-0.41)	0.34 (0.29-0.47)	<.001
Met ≥1 Leapfrog Group standard for lung, esophageal, or pancreatic resection^32^	9 (2.6)	42 (71.2)	<.001

Abbreviations: IQR, interquartile range; NA, not applicable.

^a^ Annual volume: number of procedures performed during affiliation period/number of months during affiliation period ×12 months.

^b^ Procedure volume: number of total procedures performed during full affiliation period (full study period for top-ranked hospitals).

**Figure 1.**
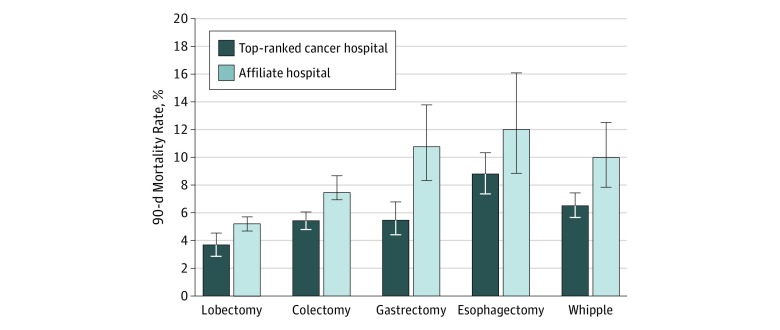
Observed 90-Day Surgical Mortality Rates by Procedure at Top-Ranked and Affiliated Hospitals Error bars indicate 95% binomial CIs. The difference in observed mortality between top-ranked hospitals and affiliates reached significance (*P* < .05) for each procedure except for esophagectomy (*P* = .08).

A total of 17 300 of 29 228 patients (59.2%; 8612 women and 8688 men; mean [SD] age, 74.7 [6.2] years) underwent complex cancer surgery at top-ranked hospitals and 11 928 (40.8%; 6287 women and 5641 men; mean [SD] age, 76.2 [6.9] years) underwent complex cancer surgery at affiliates (Table 2). Affiliated hospitals performed 318 of 1777 esophagectomies (17.9%) and 522 of 2103 gastrectomies (24.8%). The patient population cared for by affiliates was older than the population that underwent surgery at top-ranked hospitals (mean [SD] age, 76.2 [6.9] vs 74.7 [6.2] years) but otherwise clinically similar. Observed 90-day mortality was significantly higher (1.4-2.0 times higher; *P* < .001) among patients treated by affiliated hospitals compared with those treated by top-ranked hospitals for each procedure (Figure 1).

**Table 2.** Patient Characteristics

Characteristic	Patients, No. (%)		*P* Value
	Affiliated Hospital (n = 11 928)	Top-Ranked Hospital (n = 17 300)	
Age, y			
66-69	1687 (14.1)	3071 (17.8)	<.001
70-74	3119 (26.1)	5259 (30.4)	
75-79	3433 (28.8)	5254 (30.4)	
≥80	3689 (30.9)	3716 (21.5)	
Sex			
Male	5641 (47.3)	8688 (50.2)	<.001
Female	6287 (52.7)	8612 (49.8)	
Race/ethnicity			
White	10 631 (89.1)	15 031 (86.9)	<.001
Black	765 (6.4)	984 (5.7)	
Other or unknown	532 (4.5)	1285 (7.4)	
Year of surgery			
2013	1856 (15.6)	3450 (19.9)	<.001
2014	2408 (20.2)	3854 (22.3)	
2015	3521 (29.5)	4644 (26.8)	
2016	4143 (34.7)	5352 (30.9)	
Admission type			
Elective	10 955 (91.8)	16 079 (92.9)	<.001
Urgent	973 (8.2)	1221 (7.1)	
Elixhauser comorbidity score			
0	2007 (16.8)	3053 (17.6)	.01
1-2	4618 (38.7)	6853 (39.6)	
≥3	5303 (44.5)	7394 (42.7)	
Procedure			
Lobectomy	2899 (24.3)	5551 (32.1)	<.001
Colectomy	7526 (63.1)	5749 (33.2)	<.001
Gastrectomy	522 (4.4)	1581 (9.1)	<.001
Esophagectomy	318 (2.7)	1459 (8.4)	<.001
Pancreaticoduodenectomy	663 (5.6)	2960 (17.1)	<.001

### Safety of Surgery at Top-Ranked Hospitals vs Affiliated Hospitals

Risk-adjusted 90-day mortality after complex cancer surgery was significantly higher at affiliated hospitals compared with top-ranked hospitals for all 5 procedures combined (odds ratio, 1.40; 95% CI, 1.23-1.59; *P* < .001) (Table 3). The higher risk of mortality experienced by patients at affiliated hospitals ranged in magnitude when stratified by procedure, from an odds ratio of 1.32 for colectomy (95% CI, 1.12-1.56; *P* = .001) to an odds ratio of 2.04 for gastrectomy (95% CI 1.41-2.95; *P* < .001); all procedure-specific analyses were significant with the exception of esophagectomy (odds ratio, 1.48; 0.98-2.22; *P* = .06).

**Table 3.** Risk-Adjusted Odds Ratios of 90-Day Mortality at Affiliated Hospitals Compared With Top-Ranked Cancer Hospitals

Surgical Procedure	Risk-Adjusted Odds Ratio (95% CI)^a^	*P* Value
All procedures	1.40 (1.23-1.59)	<.001
Lobectomy	1.34 (1.03-1.74)	.03
Colectomy	1.32 (1.12-1.56)	.001
Gastrectomy	2.04 (1.41-2.95)	<.001
Esophagectomy	1.48 (0.98-2.22)	.06
Pancreaticoduodenectomy	1.59 (1.12-2.24)	.009

^a^ Hierarchical logistic regression for 90-day mortality adjusted for patient-level covariates (age, sex, race/ethnicity, year of surgery, Elixhauser comorbidity score, and admission type) and includes a hospital-specific random effect to account for clustering of patients within hospitals. For the model including all procedures, the model was also adjusted for type of procedure. For colectomy and gastrectomy, models also adjusted for partial or total resection. The odds ratio depicts mortality risk at affiliated hospitals with top-ranked cancer hospitals serving as the reference.

### Mortality Risk Within Each Network

**Figure 2.**
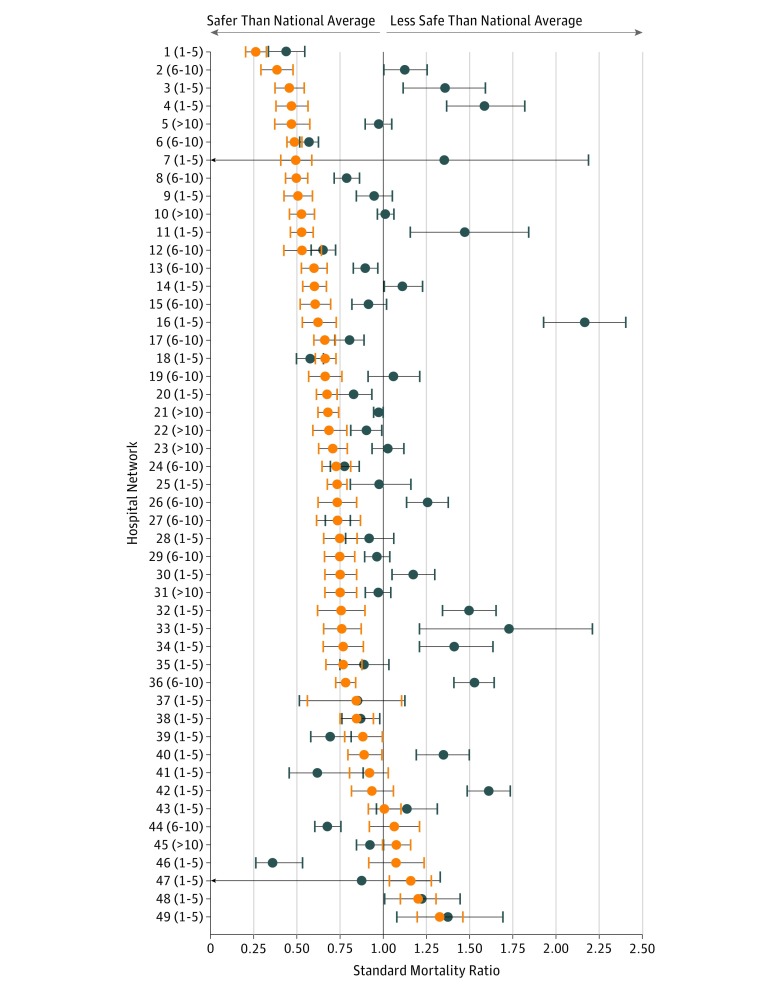
Comparison of Standardized Mortality Ratio at Top-Ranked Hospitals and Their Collective Affiliates The standardized mortality ratio (x-axis) of each top-ranked hospital is shown (orange) alongside its collective affiliates (blue) with bootstrapped 95% CIs (error bars). Hospital networks ordered by lowest top-ranked hospital standardized mortality ratio (network = 1) to highest top-ranked hospital standardized mortality ratio (network = 49), with the number of affiliated hospitals within each network in parentheses. For the number of affiliated hospitals within each network, ranges were used instead of exact values to preserve cancer hospital network confidentiality. The national mean standardized mortality ratio of 1 is based on a model including all hospitals that performed cancer surgery during the study period to avoid endogeneity.

An SMR was calculated for 49 of the top-ranked hospitals and their collective affiliates (10 networks lacked sufficient volume to reliably estimate SMR) (Figure 2). Compared with the national average, 39 of the 49 top-ranked hospitals (79.6%) and 17 of 49 collective network affiliates (34.7%) performed better than expected (SMR estimate, significantly <1). The SMR of top-ranked hospitals was lower than their collective affiliates within 41 of the 49 studied networks (83.7%; binomial 95% CI, 73.1%-93.3%), including 37 (75.5%) that reached statistical significance and 28 (57.1%) with 95% CIs that did not overlap.

When the safety of each top-ranked hospital was compared with each of its affiliates, the top-ranked hospitals outperformed 84.5% of their affiliates (290 of 343). However, low procedure volume at affiliated hospitals bias the estimates toward the national average; therefore, point estimates should be interpreted with caution (eFigure 4 in the Supplement).

### Contribution of Hospital Attributes to Differential Safety

In an attempt to explain the differential 90-day mortality risk observed between the top-ranked cancer hospitals and affiliates, several hospital attributes were individually added to the adjusted hierarchical regression model. Although no single hospital attribute eliminated the differential, the addition of annual hospital volume for the complex surgical procedures and teaching status of hospital both attenuated the magnitude and significance of the differential (eTable 5 in the Supplement).

## Discussion

This study of a large cohort of older patients receiving cancer surgery at top-ranked cancer hospitals and their network affiliates reveals that, independent of covariates, the risk of dying after complex cancer surgery is considerably higher when surgery is performed at affiliated hospitals compared with the top-ranked cancer hospitals with which they share a brand. This is not entirely surprising, as affiliated hospitals are generally smaller, less likely to be teaching hospitals, and perform complex surgical procedures with less frequency (lower volume) when compared with top-ranked hospitals.^7,8,33,34^ To this point, including hospital characteristics in adjusted models attenuated (but did not eliminate) differences in 90-day mortality.

The implications of these findings are important because previous studies suggest that affiliation status may influence hospital choice and persuade patients to assume equivalence.^19,20^ In a recent nationally representative survey of the US population, affiliation with a top-ranked cancer hospital was associated with stronger preference for complex cancer care at the affiliated hospital.^19^ In a separate study, roughly half of respondents failed to identify any differences in the safety or in the quality of care between top-ranked hospitals and their affiliates.^20^ Almost one-third of respondents who were willing to travel an additional hour to have complex cancer surgery at a top-ranked cancer hospital changed their preference in favor of a smaller local hospital if it shared an affiliation with a top-ranked cancer hospital. As a result, there is cause for concern that a proportion of the US public could misinterpret brand sharing as indicating equivalent care.

The clinical activity within these networks represented a significant (and increasing) proportion of the complex surgery performed during the study period, underscoring the potential effect of the results. By 2016, the 59 top-ranked cancer hospitals and their 343 affiliates performed 31% of the selected complex cancer surgical procedures within the Medicare population (eFigure 6 in the Supplement).

There are publicly available metrics other than *U.S. News and World Report* rankings that could support patient decision making. Annual surgical volume is one example (although prior work suggests that volume is an imperfect measure of safety).^6^ However, the current study was designed to mirror the perspective of patients, whose knowledge of specific hospital attributes (other than reputation-based ranking status) is likely limited. To some degree, the influence of hospital rankings is perpetuated by the hospitals themselves. For example, hospital ranking is listed on the website of 40 of the 50 current leading cancer hospitals (80%), while high surgical volume is alluded to in only 5 of 50 websites (10%). The current study was not designed to explain why affiliated hospitals are less safe. Our objective was to assess the differential, because a proportion of the public assumes that top-ranking hospitals and their affiliated hospitals are the same. That being said, analysis of hospital attributes, including annual surgical volume and teaching hospital status, indicates that these attributes may partially contribute to the differential mortality risk, which mirrors prior studies in complex cancer surgery.^4,7,33,35^

The perioperative safety achieved by top-ranked hospitals supports their recognition as leading cancer hospitals, as 79.6% of top-ranked hospitals performed significantly better than national average. A total of 34.7% of the affiliated networks also performed better than the national average. In the 2 instances that affiliates offered safer care than the top-ranked hospitals, the top-ranked hospitals appeared to be underperforming (SMR >1). Therefore, while surgery at the top-ranked hospitals was safer overall, some affiliates may also offer relatively safe environments for complex surgery.

The results of this study suggest an opportunity to reduce mortality through optimization within networks. The simplest concept (although challenging to implement) would be to direct the most dangerous of the complex surgical procedures to the safest hospitals within each network.^36^ Although affiliated hospitals performed 40.8% of all complex surgical procedures in the current study cohort, they performed only 17.9% of esophagectomies and 24.8% of gastrectomies, which had the largest differential in surgical mortality between affiliates and top-ranked hospitals. One could also envision using the connectivity within networks to disseminate best practices, novel surgical techniques, or even members of surgical teams to enhance the safety at smaller affiliated hospitals. Ultimately, leading cancer hospitals must assume some responsibility for leveraging relationships with their affiliated hospitals to ensure that the safety and quality of care is optimized at all hospitals that adopt their trusted brand.

### Limitations

The current study has important limitations beyond those typically ascribed to observational analysis of administrative claims. We focused on the more recognizable brands in cancer care (ie, top-ranked cancer hospitals) and their affiliates. Although we were surprised at their market share (nearly one-third of Medicare beneficiaries received complex cancer surgery within these networks), we recognize that our observations may not generalize to all scenarios in which hospitals share their brand.

The study focused on patients older than 65 years; although most complex surgical procedures occur in patients older than 65 years, and this age cohort would likely include many of the patients at higher risk for perioperative complications,^6^ it is possible that the findings could differ among cohorts of younger patients. Several clinical and sociodemographic characteristics such as tumor stage were not available and were not included in risk-adjusted models. However, multiple studies suggest that the distribution of case mix is similar across hospitals performing the same procedure, in both high-volume and low-volume settings.^4,37,38^ Although procedure-specific models were adjusted for partial and total resections for gastrectomy and colectomy, we were not able to include more granular detail of specific procedures (ie, right hemicolectomy) because the sample size within each procedure group would be too small.

We focused on hospitals that share recognized brands. In reality, networks may contain a wide array of hospital relationships (ie, limited affiliation, integrated health system, or ownership) that could affect their relative safety.^39^ However, we attempted to represent the perspective of the typical health care consumer, whose response to brand sharing most likely takes place without a detailed understanding of the nature of the hospital relationship. We did evaluate 3 attributes of the top-ranked cancer network affiliates (network size, duration of affiliation, and distance to top-ranked hospital) (eTable 6 in the Supplement) but ultimately did not identify any consistent patterns associated with 90-day mortality risk.

## Conclusions

Patients who undergo complex cancer surgery at top-ranked cancer hospitals are associated with a considerably lower risk of mortality within 90 days than those having surgery at their affiliate hospitals. This information may affect hospital preference for a subset of patients, as previous work suggests that a large fraction of the general public equates brand sharing with equivalent care within top-ranked networks.^19,20,40^ Further investigation of performance across trusted cancer networks could enhance informed decision making for complex cancer care.

## Notes

Supplement. **eAppendix.** Supplemental Methods **eFigure 1.** Hospital Selection **eFigure 2.** Comparison of SMRs at Top-Ranked Hospitals and Their Collective Affiliates Within Each Network, Excluding Colectomy **eFigure 3.** Comparison of Risk-Standardized Mortality Ratio (RSMR) Within Networks Between Top-Ranked Hospitals and Their Set of Collective Affiliates **eFigure 4.** Comparison of Risk-Standardized Mortality Ratio (RMSR) Within Networks Between Each Top-Ranked Hospital and Each of its Affiliates **eFigure 5.** Comparison of SMRs at Top-Ranked Hospitals and Their Collective Affiliates by Quintile of Best Hospitals for Cancer, *U.S. News and World Report* Top 50 Rankings (2015 Data) **eFigure 6.** Proportion of Surgery Performed Among Cancer Networks Included in Analysis (Top-Ranked Hospitals [n=59] and Their Affiliates [n=343]) by Year **eTable 1.** *International Classification of Diseases, Ninth Revision* (ICD-9) and *International Classification of Diseases, Tenth Revision* (ICD-10) Diagnosis and Procedure Codes Used to Identify the Study Population **eTable 2.** Risk-Adjusted 30-Day Mortality at Affiliate Hospitals Compared With Top-Ranked Hospitals **eTable 3.** Risk-Adjusted 90-Day Mortality at Affiliate Hospitals Compared With Top-Ranked Cancer Hospitals, Excluding Colectomy **eTable 4.** Risk-Adjusted 90-day Mortality at Affiliate Hospitals Compared With Top-Ranked Cancer Hospitals, Excluding Patients Receiving Surgery at the Two Largest Networks (Top-Ranked Hospitals and Their Affiliates) **eTable 5.** Association of Select Hospital Attributes With Risk-Adjusted 90-Day Mortality **eTable 6.** Summary of Attributes Among Affiliate Hospitals (N = 343) and Association With Risk-Adjusted 90-Day Mortality **eReferences**
