# Data Error in Results

**DOI:** 10.1001/jamanetworkopen.2019.9327

**Published:** 2019-09-13

In the Original Investigation titled “Differential Safety Between Top-Ranked Cancer Hospitals and Their Affiliates for Complex Cancer Surgery,”^1^ published April 12, 2019, there was a data error in the Results section. In the second paragraph of the Mortality Risk Within Each Network subsection, the first sentence should have read “When the safety of each top-ranked hospital was compared with each of its affiliates, the top-ranked hospitals outperformed 84.3% of their affiliates (289 of 343).” This article has been corrected.^1^
