# Supplementary material for: Differential Safety Between Top-Ranked Cancer Hospitals and Their Affiliates for Complex Cancer Surgery
Source: JAMA Netw Open. 2019 Apr 12;2(4):e191912. doi: 10.1001/jamanetworkopen.2019.1912 (PMC6481444; doi:10.1001/jamanetworkopen.2019.1912)
Supplement: Supplement. — eAppendix. Supplemental Methods eFigure 1. Hospital Selection eFigure 2. Comparison of SMRs at Top-Ranked Hospitals and Their Collective Affiliates Within Each Network, Excluding Colectomy eFigure 3. Comparison of Risk-Standardized Mortality Ratio (RSMR) Within Networks Between Top-Ranked Hospitals and Their Set of Collective Affiliates eFigure 4. Comparison of Risk-Standardized Mortality Ratio (RMSR) Within Networks Between Each Top-Ranked Hospital and Each of its Affiliates eFigure 5. Comparison of SMRs at Top-Ranked Hospitals and Their Collective Affiliates by Quintile of Best Hospitals for Cancer, U.S. News and World Report Top 50 Rankings (2015 Data) eFigure 6. Proportion of Surgery Performed Among Cancer Networks Included in Analysis (Top-Ranked Hospitals [n=59] and Their Affiliates [n=343]) by Year eTable 1. International Classification of Diseases, Ninth Revision (ICD-9) and International Classification of Diseases, Tenth Revision (ICD-10) Diagnosis and Procedure Codes Used to Identify the Study Population eTable 2. Risk-Adjusted 30-Day Mortality at Affiliate Hospitals Compared With Top-Ranked Hospitals eTable 3. Risk-Adjusted 90-Day Mortality at Affiliate Hospitals Compared With Top-Ranked Cancer Hospitals, Excluding Colectomy eTable 4. Risk-Adjusted 90-day Mortality at Affiliate Hospitals Compared With Top-Ranked Cancer Hospitals, Excluding Patients Receiving Surgery at the Two Largest Networks (Top-Ranked Hospitals and Their Affiliates) eTable 5. Association of Select Hospital Attributes With Risk-Adjusted 90-Day Mortality eTable 6. Summary of Attributes Among Affiliate Hospitals (N = 343) and Association With Risk-Adjusted 90-Day Mortality eReferences [file jamanetwopen-2-e191912-s001.pdf]

Supplementary Online Content

Hoag JR, Resio BJ, Monsalve AF, et al. Differential safety between top-ranked cancer hospitals and their affiliates for complex cancer surgery. *JAMA Netw Open.* 2019;2(4):e191912. doi:10.1001/jamanetworkopen.2019.1912

eAppendix. Supplemental Methods
eFigure 1. Hospital Selection
eFigure 2. Comparison of SMRs at Top-Ranked Hospitals and Their Collective Affiliates Within Each Network, Excluding Colectomy
eFigure 3. Comparison of Risk-Standardized Mortality Ratio (RSMR) Within Networks Between Top-Ranked Hospitals and Their Set of Collective Affiliates
eFigure 4. Comparison of Risk-Standardized Mortality Ratio (RMSR) Within Networks Between Each Top-Ranked Hospital and Each of its Affiliates
eFigure 5. Comparison of SMRs at Top-Ranked Hospitals and Their Collective Affiliates by Quintile of Best Hospitals for Cancer, *U.S. News and World Report* Top 50 Rankings (2015 Data)
eFigure 6. Proportion of Surgery Performed Among Cancer Networks Included in Analysis (Top-Ranked Hospitals [n=59] and Their Affiliates [n=343]) by Year
eTable 1. *International Classification of Diseases, Ninth Revision* (ICD-9) and *International Classification of Diseases, Tenth Revision* (ICD-10) Diagnosis and Procedure Codes Used to Identify the Study Population
eTable 2. Risk-Adjusted 30-Day Mortality at Affiliate Hospitals Compared With Top-Ranked Hospitals
eTable 3. Risk-Adjusted 90-Day Mortality at Affiliate Hospitals Compared With Top-Ranked Cancer Hospitals, Excluding Colectomy
eTable 4. Risk-Adjusted 90-day Mortality at Affiliate Hospitals Compared With Top-Ranked Cancer Hospitals, Excluding Patients Receiving Surgery at the Two Largest Networks (Top-Ranked Hospitals and Their Affiliates)
eTable 5. Association of Select Hospital Attributes With Risk-Adjusted 90-Day Mortality
eTable 6. Summary of Attributes Among Affiliate Hospitals (N = 343) and Association With Risk-Adjusted 90-Day Mortality
eReferences

This supplementary material has been provided by the authors to give readers additional information about their work.

eAppendix. Supplemental Methods

Eligible patients undergoing non-emergent complex cancer surgery were included in the study population if their procedure was performed at either 1) a top-ranked cancer hospital or 2) a hospital with evidence of a brand-sharing affiliation to a top-ranked cancer hospital during the 45-month study period (January 1, 2013 to October 1, 2016). Top-ranked cancer hospitals were identified from the US World and News Report™ Rankings: Best Hospitals for Cancer and were defined as hospitals that were ranked among the "Top 50" cancer hospitals at least once between 2013 and 2016. Regardless of how many times a hospital was ranked in the top 50 between 2013 and 2016, it was included as a top-ranked hospital for the entire study period because its positive reputation most likely preceded or exceeded the duration of the top 50 ranking status.[1] In a few instances, a top-ranked cancer hospital also qualified as an affiliate of another top-ranked hospital. In each of these few instances, the hospital shared the same name as a higher-ranked top hospital and had no established affiliates of its own. Therefore, it was counted as an affiliate hospital only. A total of 59 top-ranked cancer hospitals were included.

Affiliate hospitals were identified in two steps. First, the American Hospital Association (AHA) Annual Survey Database was queried from 2012-2015 to identify hospitals that participated in a shared network or system with top-ranked cancer hospital. This step identified 637 candidate affiliates. Second, brand-sharing was established between the affiliate and the top-ranked cancer hospital. Specifically, it was confirmed that the name of the top-ranked hospital was publicly associated with the affiliate hospital, as opposed to more restricted relationships that were not strategically promoted (i.e. financial only). To establish brand-sharing, each of the 637 candidate affiliates were evaluated for online evidence in the form of advertising on the hospital website (homepage, cancer center page, "About Us" page, etc) or in the form of an online news publication. A total of 388 affiliate hospitals were identified as brand-sharing with a top-ranked cancer hospital: 367 through website advertising on the hospital homepage or cancer center page, and 21 through website evidence on a page other than the homepage or cancer center page (i.e. "About Us" page) or from another source (i.e. news publication). Four candidate affiliate hospitals were subsequently excluded due to sharing the same CMS Certification Number (CCN) (MEDPAR Provider Number) with a top-ranked cancer hospital.

For each of 384 eligible affiliate hospitals with evidence of brand-sharing with a top-ranked cancer hospital, the start date of affiliation and the presence of the affiliation at any time during the study period were confirmed. Dates of affiliation were confirmed from three primary sources: hospital website, news publication, or a direct call to the hospital public relations department. Not all hospital affiliations were continuous during the study period. Therefore, analysis was limited to patients receiving care during the affiliation period with a top-ranked cancer hospital. In addition, hospital affiliations beginning after January 31, 2016 were excluded due to insufficient clinical activity to evaluate 90-day mortality (n=3 hospitals). Finally, hospitals that did not perform complex cancer surgery (lobectomy, colectomy, gastrectomy, esophagectomy, or Whipple) during the study period were excluded (n=38 hospitals). A total of 59 top-ranked cancer hospitals and 343 affiliate hospitals were included in analysis.

Hospital characteristics included from the AHA Annual Survey Database were bed size, Commission on Cancer (CoC) accredited cancer program, and teaching status. Additional hospital attributes included duration in years of affiliation to a top-ranked cancer hospital (affiliates only), average annual procedure volume, proportion of minimally invasive colectomies, and whether the hospital had met the surgical volume standard for least one type of resection for cancer recommended by the Leapfrog Group: esophageal, lung, or pancreatic.[2]

As further testament to the reputation of our "top-ranked" hospital cohort, there was greater than 90% overlap between U.S. News and World Report™ and Becker's Hospital Review™, with 56 of 59 of our top-ranked hospitals appearing in Becker's Top 100 Cancer hospitals in 2016. Additionally, many easily accessible (via Google Search for 'top-ranked cancer hospitals') public ranking sources of "best cancer hospitals" closely mirror or are based on U.S. News and World Report ™ rankings: (i.e. Medscape, CNN, Ranker, Livestrong, Men's Health, Statista, AdvisoryHQ, Know Cancer, Very Well Health).

© 2019 Hoag JR et al. *JAMA Network Open.*

eFigure 1. Hospital Selection

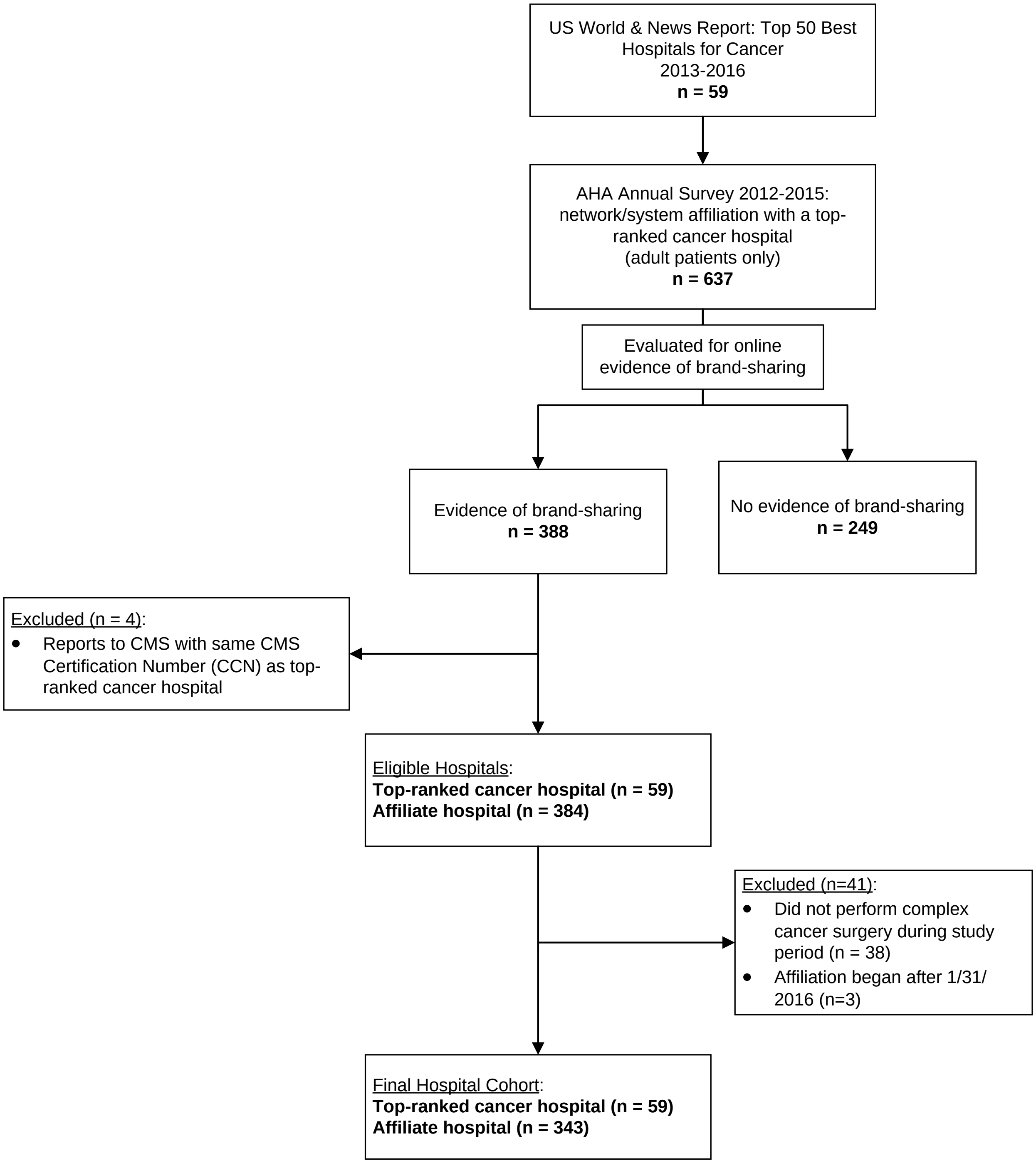

© 2019 Hoag JR et al. *JAMA Network Open.*

eFigure 2. Comparison of SMRs at Top-Ranked Hospitals and Their Collective Affiliates Within Each Network, Excluding Colectomy

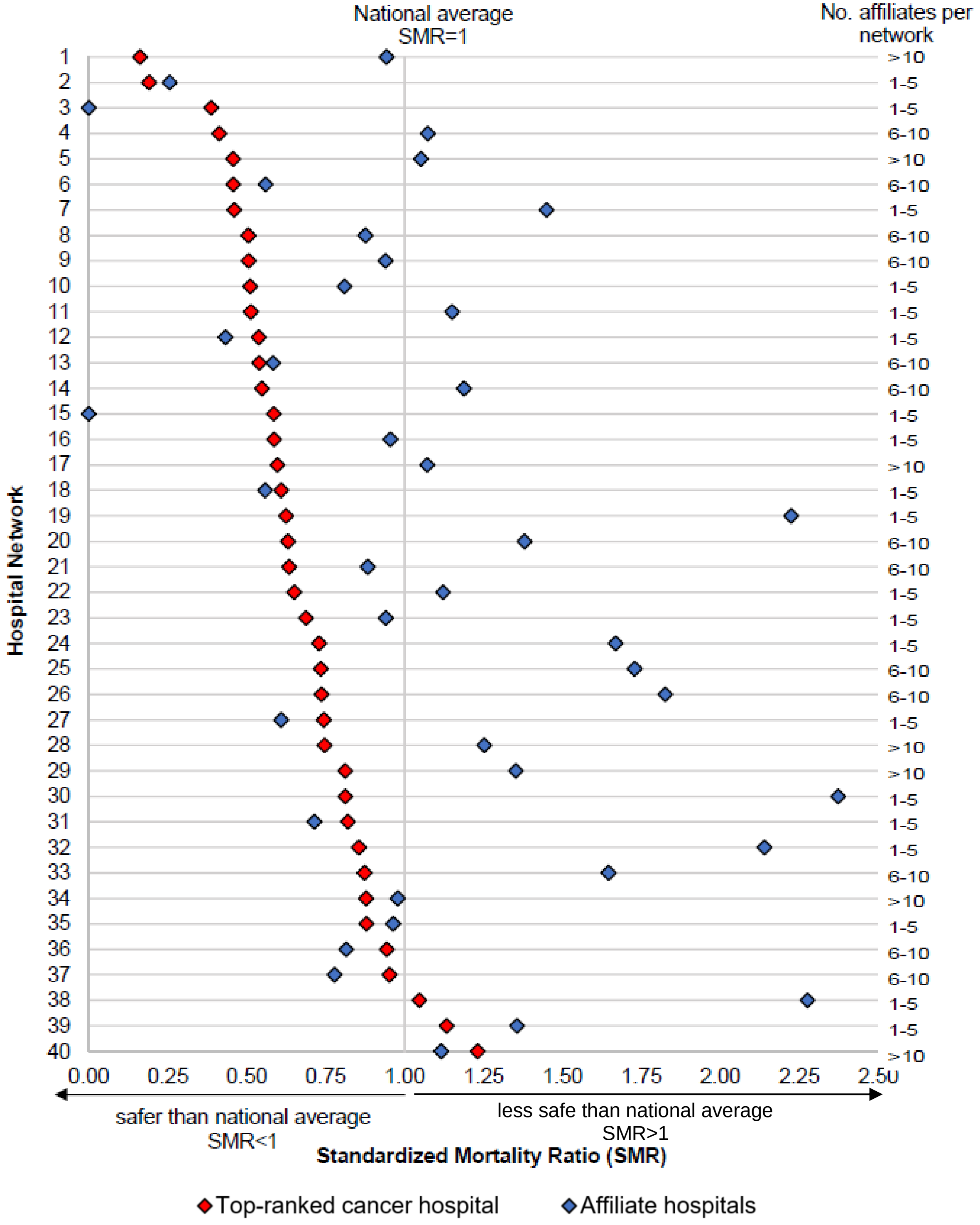

Note: Hospital networks ordered by lowest top-ranked hospital SMR (network=1) to highest top-ranked hospital SMR (network=40). Column on the right indicates the number of affiliate hospitals within each network, with ranges instead of exact values to preserve cancer hospital network confidentiality. National average of SMR = 1 is based on a model including all hospitals that performed cancer surgery during the study period to avoid endogeneity.

© 2019 Hoag JR et al. *JAMA Network Open.*

eFigure 3. Comparison of Risk-Standardized Mortality Ratio (RSMR) Within Networks Between Top-Ranked Hospitals and Their Set of Collective Affiliates

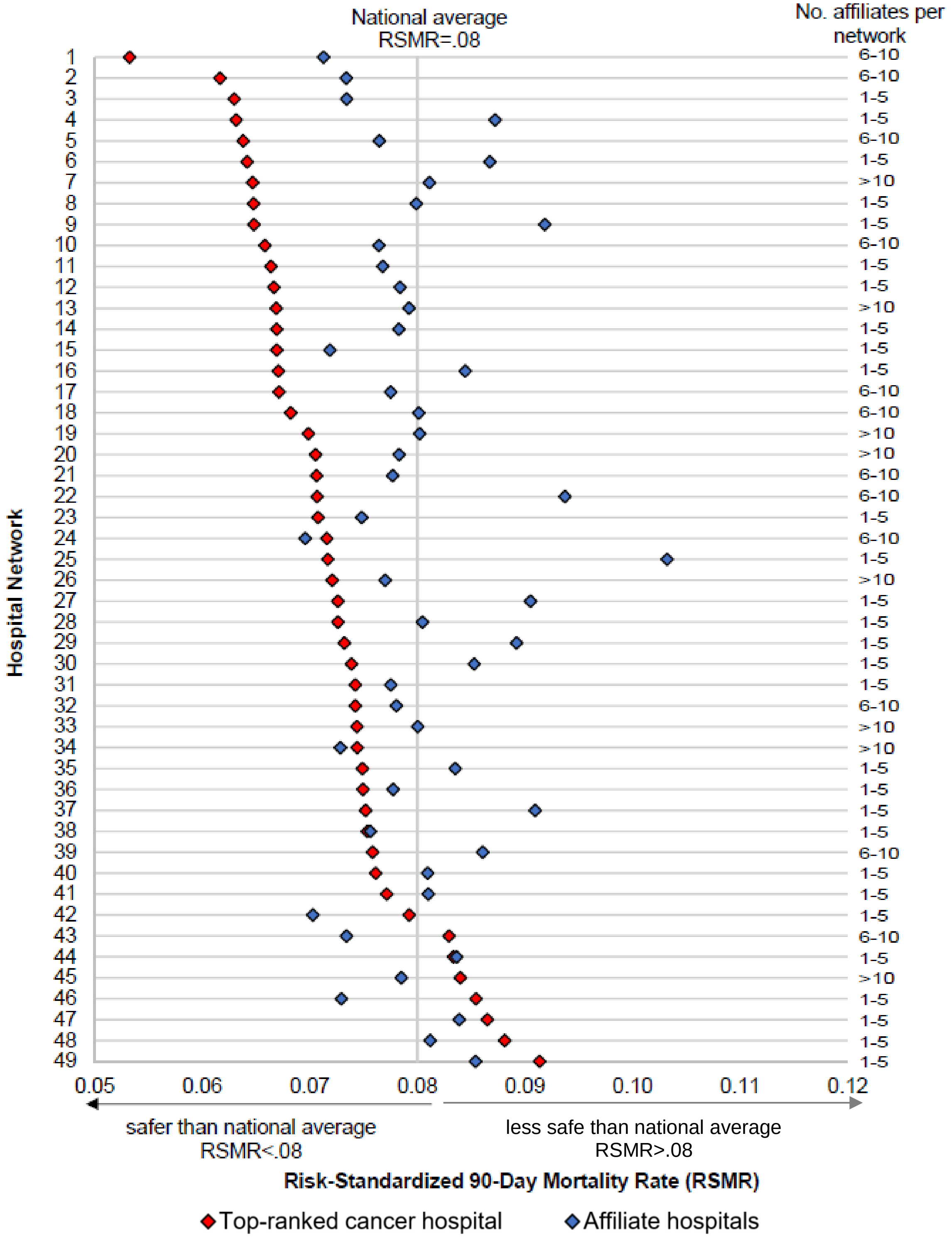

Note: Hospital networks ordered by lowest top-ranked hospital RSMR (network=1) to highest top-ranked hospital RSMR (network=49). Column on the right indicates the number of affiliate hospitals within each network, with ranges instead of exact values to preserve cancer hospital network confidentiality. National average of RSMR = .08 is based on a model including all hospitals that performed cancer surgery during the study period to avoid endogeneity.

© 2019 Hoag JR et al. *JAMA Network Open.*

eFigure 4. Comparison of Risk-Standardized Mortality Ratio (RMSR) Within Networks Between Each Top-Ranked Hospital and Each of its Affiliates

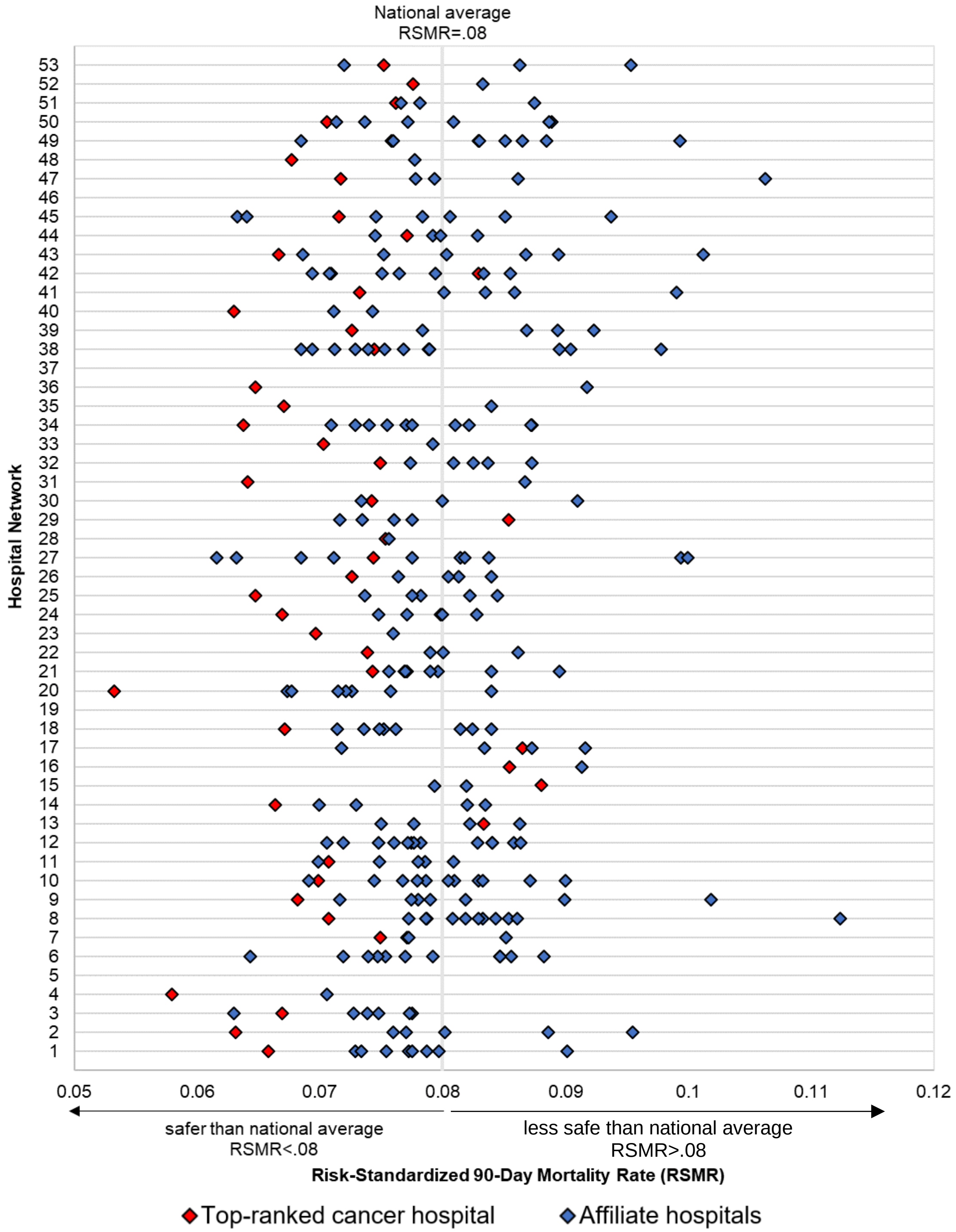

Note: Hospital networks with unique number of affiliate hospitals are excluded from the figure to preserve cancer hospital network confidentiality (n=4 cancer networks excluded). National average of RSMR = .08 is based on a model including all hospitals that performed cancer surgery during the study period to avoid endogeneity.

© 2019 Hoag JR et al. *JAMA Network Open.*

eFigure 5. Comparison of SMRs at Top-Ranked Hospitals and Their Collective Affiliates by Quintile of Best Hospitals for Cancer, *U.S. News and World Report* Top 50 Rankings (2015 Data)

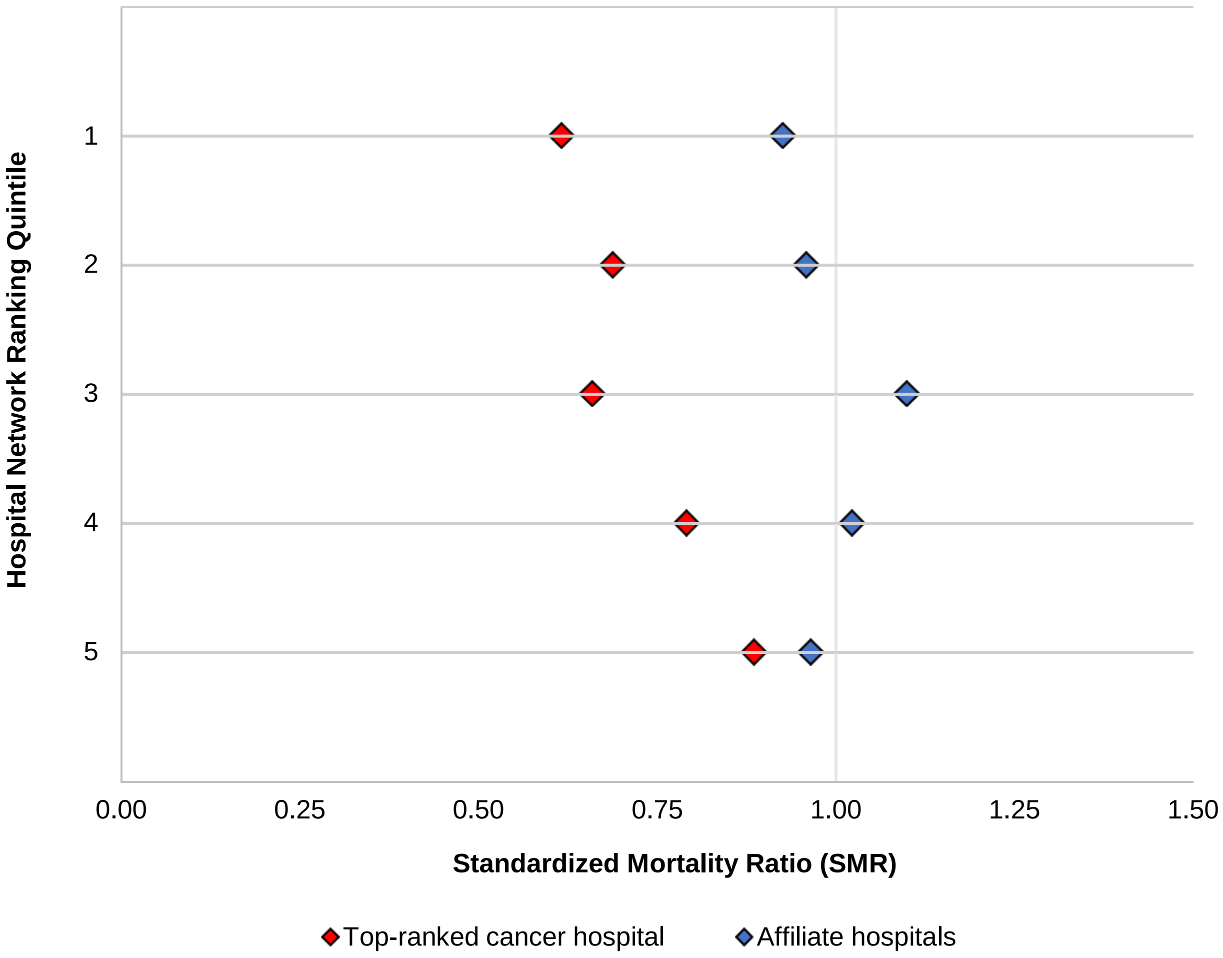

Note: Hospital networks ordered by ranking quintiles, defined using the Top 50 Best Hospitals for cancer, U.S. News and World Report ranking from 2015. Quintile 1 represents the top 10 hospitals; quintile 2 represents hospitals ranked 11-20; quintile 3 represents hospitals ranked 21-30; quintile 4 represents hospitals ranked 31-40; and quintile 5 represents hospitals ranked 41-50. Hospitals that did not appear in the Top 50 list of best hospitals for cancer in 2015 but appeared in the Top 50 for 2013, 2014, or 2016 were included in the lowest quintile of hospitals, ranked 41-50.

© 2019 Hoag JR et al. *JAMA Network Open.*

eFigure 6. Proportion of Surgery Performed Among Cancer Networks Included in Analysis (Top-Ranked Hospitals [n=59] and Their Affiliates [n=343]) by Year

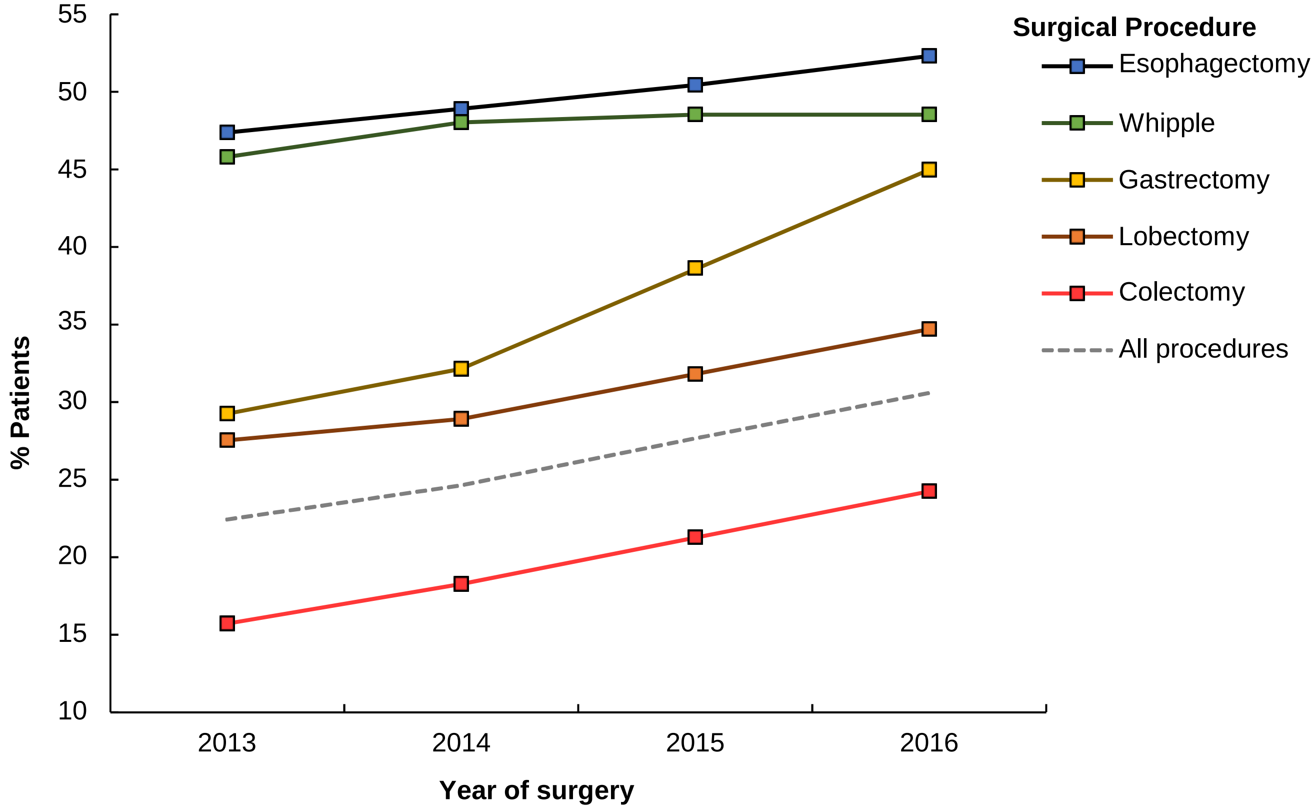

Note: Estimates are derived for each procedure as the proportion of all eligible complex cancer surgeries among Medicare beneficiaries during the study period performed at top-ranked and affiliate hospitals.

© 2019 Hoag JR et al. *JAMA Network Open.*

eTable 1. *International Classification of Diseases, Ninth Revision* (ICD-9) and *International Classification of Diseases, Tenth Revision* (ICD-10) Diagnosis and Procedure Codes Used to Identify the Study Population

Diagnosis	*ICD-9*	*ICD-10*	Procedure	*ICD-9*	*ICD-10*
Lung cancer	162, 162.0, 162.2, 162.3, 162.4, 162.5, 162.8, 162.9	C33, C34, C34.0, C34.00, C34.01, C34.02, C34.1, C34.10, C34.11, C34.12, C34.2, C34.3, C34.30, C34.31, C34.32, C34.8, C34.80, C34.81, C34.82, C34.9, C34.90, C34.91, C34.92	Lobectomy	32.4, 32.41, 32.49	0BTC4ZZ, 0BTD4ZZ, 0BTF4ZZ, 0BTG4ZZ, 0BTJ4ZZ, 0BTC0ZZ, 0BTD0ZZ, 0BTF0ZZ, 0BTG0ZZ, 0BTJ0ZZ
Colon cancer	153, 153.0, 153.1, 153.2, 153.3, 153.4, 153.5, 153.6, 153.7, 153.8, 153.9	C18, C18.0, C18.1, C18.2, C18.3, C18.4, C18.5, C18.6, C18.7, C18.8, C18.9	Partial Colectomy	45.73, 45.74, 45.75, 45.76, 45.79, 17.3, 17.31, 17.32, 17.33, 17.34, 17.35, 17.39	0DTF0ZZ, 0DTK0ZZ, 0DTP0ZZ, 0DTG0ZZ, 0DTL0ZZ, 0DTM0ZZ, 0DTN0ZZ, 0DBF0ZZ, 0DBK0ZZ, 0DBP0ZZ, 0DBG0ZZ, 0DBL0ZZ, 0DBM0ZZ, 0DBN0ZZ, 0DTF4ZZ, 0DTK4ZZ, 0DTP4ZZ, 0DTG4ZZ, 0DTL4ZZ, 0DTM4ZZ, 0DTN4ZZ, 0DBF4ZZ, 0DBK4ZZ, 0DBP4ZZ, 0DBG4ZZ, 0DBL4ZZ, 0DBM4ZZ, 0DBN4ZZ
			Total Colectomy	45.8, 45.81, 45.82, 45.83	0DTE0ZZ, 0DTE4ZZ
Esophageal cancer	150, 150.1, 150.2, 150.3, 150.4, 150.5, 150.8, 150.9	C15, C15.3, C15.4, C15.5, C15.8, C15.9	Esophagectomy	42.4, 42.40, 42.41, 42.42, 43.99	0DT10ZZ, 0DT20ZZ, 0DT30ZZ, 0DT40ZZ, 0DT50ZZ, 0DT14ZZ, 0DT24ZZ, 0DT34ZZ, 0DT44ZZ, 0DT54ZZ, 0DB10ZZ, 0DB20ZZ, 0DB30ZZ, 0DB40ZZ, 0DB50ZZ, 0DB14ZZ, 0DB24ZZ, 0DB34ZZ, 0DB44ZZ, 0DB54ZZ
Gastric cancer	151, 151.1, 151.2, 151.3, 151.4, 151.5, 151.6, 151.8, 151.9	C16, C16.0, C16.1, C16.2, C16.3, C16.4, C16.5, C16.6, C16.8, C16.9	Partial Gastrectomy	43.5, 43.6, 43.7, 43.8,	0DB60ZZ, 0DB70ZZ, 0DB64ZZ, 0DB74ZZ
			Total Gastrectomy	43.9, 43.91, 43.99	0DT60ZZ, 0DT70ZZ, 0DT64ZZ, 0DT74ZZ

© 2019 Hoag JR et al. *JAMA Network Open.*

Diagnosis	*ICD-9*	*ICD-10*	Procedure	*ICD-9*	*ICD-10*
Pancreatic cancer	157, 157.1, 157.2, 157.3, 157.4, 157.8, 157.9	C25, C25.0, C25.1, C25.2, C25.3, C25.4, C25.7, C25.8, C25.9	Whipple	52.51, 52.6, 52.7, 52.53	0FTG4ZZ, 0FTG0ZZ<br>+<br>0DT94ZZ, 0DT90ZZ<br>or<br>0FBG4ZZ, 0FBG0ZZ<br>+<br>0DB94ZZ, 0DB90ZZ

© 2019 Hoag JR et al. *JAMA Network Open.*

eTable 2. Risk-Adjusted 30-Day Mortality at Affiliate Hospitals Compared With Top-Ranked Hospitals[a]

Surgical procedure	Risk-adjusted Odds Ratio (95% CI)	*P* value
All procedures	1.46 (1.23 to 1.72)	<.001
Lobectomy	1.56 (1.14 to 2.14)	.005
Colectomy	1.42 (1.12 to 1.81)	.004
Gastrectomy	2.13 (1.30 to 3.49)	.003
Esophagectomy	1.06 (0.58 to 1.91)	.86
Whipple	1.69 (1.05 to 2.72)	.03

[a] Hierarchical logistic regression for 30-day mortality adjusted for patient-level covariates (age, sex, race, year of surgery, Elixhauser comorbidity score, admission type) and includes a hospital-specific random effect. For the model with all procedures, the model was also adjusted for type of procedure. For colectomy and gastrectomy, models also adjusted for partial or total resection. The odds ratio depicts mortality risk at affiliate hospitals with top-ranked cancer hospitals serving as the reference.

© 2019 Hoag JR et al. *JAMA Network Open.*

eTable 3. Risk-Adjusted 90-Day Mortality at Affiliate Hospitals Compared With Top-Ranked Cancer Hospitals, Excluding Colectomy[a]

**Surgical procedure**	**Risk-adjusted Odds Ratio (95% CI)**	***P* value**
All procedures excluding colectomy	1.49 (1.25 to 1.77)	< .001

[a] Hierarchical logistic regression for 90-day mortality adjusted for patient-level covariates (age, sex, race, year of surgery, Elixhauser comorbidity score, admission type, procedure) and includes a hospital-specific random effect. The odds ratio depicts mortality risk at affiliate hospitals with top-ranked cancer hospitals serving as the reference.

© 2019 Hoag JR et al. *JAMA Network Open.*

eTable 4. Risk-Adjusted 90-day Mortality at Affiliate Hospitals Compared With Top-Ranked Cancer Hospitals, Excluding Patients Receiving Surgery at the Two Largest Networks (Top-Ranked Hospitals and Their Affiliates)[a]

**Surgical procedure**	**Risk-adjusted Odds Ratio (95% CI)**	***P* value**
All procedures	1.40 (1.22 to 1.61)	<.001
Lobectomy	1.42 (1.06 to 1.90)	.018
Colectomy	1.28 (1.07 to 1.53)	.008
Gastrectomy	1.98 (1.29 to 3.03)	.002
Esophagectomy	1.72 (1.04 to 2.84)	.03
Whipple	1.86 (1.23 to 2.81)	.003

[a] Hierarchical logistic regression for 90-day mortality adjusted for patient-level covariates (age, sex, race, year of surgery, Elixhauser comorbidity score, admission type) and includes a hospital-specific random effect. For the model with all procedures, the model was also adjusted for type of procedure. For colectomy and gastrectomy, models also adjusted for partial or total resection. The odds ratio depicts mortality risk at affiliate hospitals with top-ranked cancer hospitals serving as the reference.

© 2019 Hoag JR et al. *JAMA Network Open.*

eTable 5. Association of Select Hospital Attributes With Risk-Adjusted 90-Day Mortality[a]

**Hospital attribute**	**Risk-adjusted Odds Ratio (95% CI)**	***P* value**
No *Hospital* Attributes added<br>(i.e. primary model reported in body of manuscript)	1.40 (1.23 to 1.59)	<.001
Hospital Bed Size	1.28 (1.09 to 1.50)	.003
CoC accreditation status[b]	1.40 (1.23 to 1.59)	<.001
Teaching status of hospital	1.23 (1.03 to 1.48)	.03
Annual procedure volume	1.22 (1.05 to 1.42)	.010
Proportion of cases performed by MIS techniques[c]	1.36 (1.20 to1.55)	<.001
Meeting Leapfrog Group minimum volume standard[d]	1.32 (1.12 to 1.57)	.001

[a] Hierarchical logistic regression for 90-day mortality adjusted for patient-level covariates (age, sex, race, year of surgery, Elixhauser comorbidity score, admission type, procedure) and includes a hospital-specific random effect. The odds ratio depicts mortality risk at affiliate hospitals with top-ranked cancer hospitals serving as the reference.

[b] CoC = Commission on Cancer, a program of the American College of Surgeons

[c] MIS: minimally invasive surgery; calculated for colectomy only for entire study period

[d] Whether or not each hospital met the surgical volume standard for least one type of resection for cancer recommended by the Leapfrog Group: esophageal, lung, or pancreatic.[2]

© 2019 Hoag JR et al. *JAMA Network Open.*

eTable 6. Summary of Attributes Among Affiliate Hospitals (N = 343) and Association With Risk-Adjusted 90-Day Mortality

Affiliate attribute	N (%)	Risk-adjusted Odds Ratio (95% CI)[a]	*P* value
**Network size[b]**			
1-5	101 (29.4)	ref	--
6-10	105 (30.6)	0.81 (0.65 to 1.01)	.06
>10	137 (40.0)	0.90 (0.73 to 1.10)	.29
**Duration of affiliation (years)**			
≤1-1.9	71 (20.7)	ref	--
2-2.9	46 (13.4)	1.23 (0.86 to 1.77)	.26
≥3	226 (65.9)	1.18 (0.90 to 1.55)	.24
**Distance from top-ranked hospital (miles)**			
<1-25	89 (26.0)	ref	--
26-100	131 (38.2)	1.35 (1.09 to 1.67)	.006
101-500	55 (16.0)	1.22 (0.94 to 1.58)	.14
>500	68 (19.8)	1.18 (0.94 to 1.50)	.15

[a] Hierarchical logistic regression for 90-day mortality adjusted for patient-level covariates (age, sex, race, year of surgery, Elixhauser comorbidity score, admission type, procedure) and includes a hospital-specific random effect. Model estimated for patients treated at affiliate hospitals only.
[b] Total number of affiliate hospitals in the network

© 2019 Hoag JR et al. *JAMA Network Open.*

eReferences

1. Sehgal AR: The role of reputation in US News & World Report's rankings of the top 50 American hospitals. Annals of internal medicine 152:521-525, 2010

2. Surgical Volume. *The Leapfrog Group* http://www.leapfroggroup.org/ratings-reports/surgical-volume. Accessed August 2, 2018.
